# Is speech enough? Language development evaluation in the first 6 years of prelingually deaf children. A systematic review

**DOI:** 10.1590/2317-1782/20232022084en

**Published:** 2023-11-10

**Authors:** Raúl Francisco Lara Barba, Yadira Niyireth Angamarca Guanuche, Andrea Lorena Mera Herrera, Erick Fabricio Gudiño Chinchin, Victor Hugo Altamirano Sampedro, María Belén Mena Ayala

**Affiliations:** 1 Facultad de Ciencias de la Discapacidad, Atención pre-hospitalaria y Desastres, Universidad Central del Ecuador ­- UCE - Quito (Pichincha), Ecuador.; 2 Universidad Central del Ecuador - UCE - Quito (Pichincha), Ecuador.

**Keywords:** Deafness, Hearing Loss, Language, Child Language, Disability Evaluation, Deafness, Hearing Loss, Language, Child Language, Disability Evaluation, Deafness, Hearing Loss, Language, Child Language, Disability Evaluation, Deafness, Hearing Loss, Language, Child Language, Disability Evaluation, Deafness, Hearing Loss, Language, Child Language, Disability Evaluation

## Abstract

**Purpose:**

To synthesize the relevant scientific information regarding the assessment of language development in prelingually deaf children during their first six years of life, in order to determine whether it is sufficient to confirm the presence of some language development in this population, or if a more integrated approach would be more appropriate.

**Research strategies:**

A structured review of the relevant scientific literature was carried out in the following databases: PubMed, Lilacs, Ibecs, Trip DataBase, Cochrane library, Clinical Trial and Nice.

**Selection criteria:**

Systematic reviews, health technology assessments, randomized clinical trials, observational cohorts and case-control studies; including publications of assessments concerning any aspect of language development or any intervention in any language. Evaluations related exclusively to hearing and speech, to central, sudden or transient deafness, to deaf-blindness, to further disabilities or to autism spectrum disorders, were excluded.

**Data analysis:**

GRADE methodology was used to analyze evidence quality.

**Results:**

It is possible to evaluate the language development of prelingually deaf children. A moderate quality of evidence was obtained, suggesting that the evaluations’ results are fairly trustworthy, provided that the assessments are conducted within an integrated approach of other linguistic elements.

**Conclusion:**

The results of the language evaluations must be supported mostly by receptive and expressive language data, and the found evidence can be improved by combining the assessments of the formal linguistic elements of both oral and gestural modalities with the pragmatic components of the communication process.

## INTRODUCTION

In many ways, the investigation of prelingual deafness in children has been a difficult issue to consider, because most of its approaches arise from the physical medicine and rehabilitation perspective, along with an oralistic view of the physical component of deafness: the hearing impairment^(1)^. In other words, the urge, when facing a disability or a hearing impairment condition, to try to rehabilitate the child so they can “speak”, by any means necessary. Those who adhere to this idea start from the hypothesis that hearing is enough to fully develop language^(2)^. Hence, it is necessary to consider whether the assessment of hearing and speech production is considered sufficient to establish some language development in deaf children, or if a more integrated approach would be more strategic^(3)^.

The first six years of every child, deaf or hearing, are crucial to determine their future development, including language. During the first three years occurs the phenomena of natural language acquisition, and in the subsequent three years this assimilation is consolidated, so that from six years old onward the processes of organized and planned language learning may take place^(4)^. However, the aforementioned sequence of stages is not always applicable to situations where the language might not adhere to the oral modality. Therefore, a lack of follow-up leads to significant language development risks that could be prevented by seeking the guidance of professionals specialized in this population^(3)^. The present review targets the population of prelingually deaf children with multifactorial hearing loss, as well as its consequences, such as the substantial negative impact on the developing auditory system and on language development, the risk of delayed speech (difficulty to produce sounds and/or to understand speech), poor academic performance, behavioral problems, and decreased quality of life^(5)^.

When word production and speech are not sufficient to predict any language development, we must necessarily resort to other options. In the seventies, several philosophical, linguistic, and sociological movements were promoted to support a broader understanding of language, from functionality to pragmatics, that is, children's language had to be interpreted starting at the communication aspect, taking into consideration the intention and the situation in which the child assimilates their input^(6)^. Deaf children are not outside of this conjecture. That being said, an extra factor happens to them: the world is built for listeners. If their development is directed to the oral sphere, their acquisition process is not spontaneous and natural, but rather a difficult learning process planned by an adult; if their development is gestural, it is usually natural, but with limitations related to the environment in which they are born^(4)^. A complete language assessment is considered a complex task to perform, given the assortment of multiple components that differ in their conceptualization according to the chosen perspective. In general terms, language can combine elements of production and comprehension, being influenced by elements of hearing, phonation, form (phonology and morphology), content (semantics), and use (pragmatics)^(7)^.

The conception of language and language development is undoubtedly broad and surpasses hearing and producing words. Nevertheless, the everyday reality seems to keep pushing the fate of deaf children towards the search for an apparently “normal” hearing, without considering other options. For this reason, the present systematic review seeks to answer the following research question: What language development assessments are currently being performed on prelingually deaf children during their first 6 years of life?

## OBJECTIVE

To identify, select, evaluate, and synthesize the relevant evidence available on the current assessment of language development during the first six years of prelingually deaf children.

## RESEARCH STRATEGY

**Figure 1:**
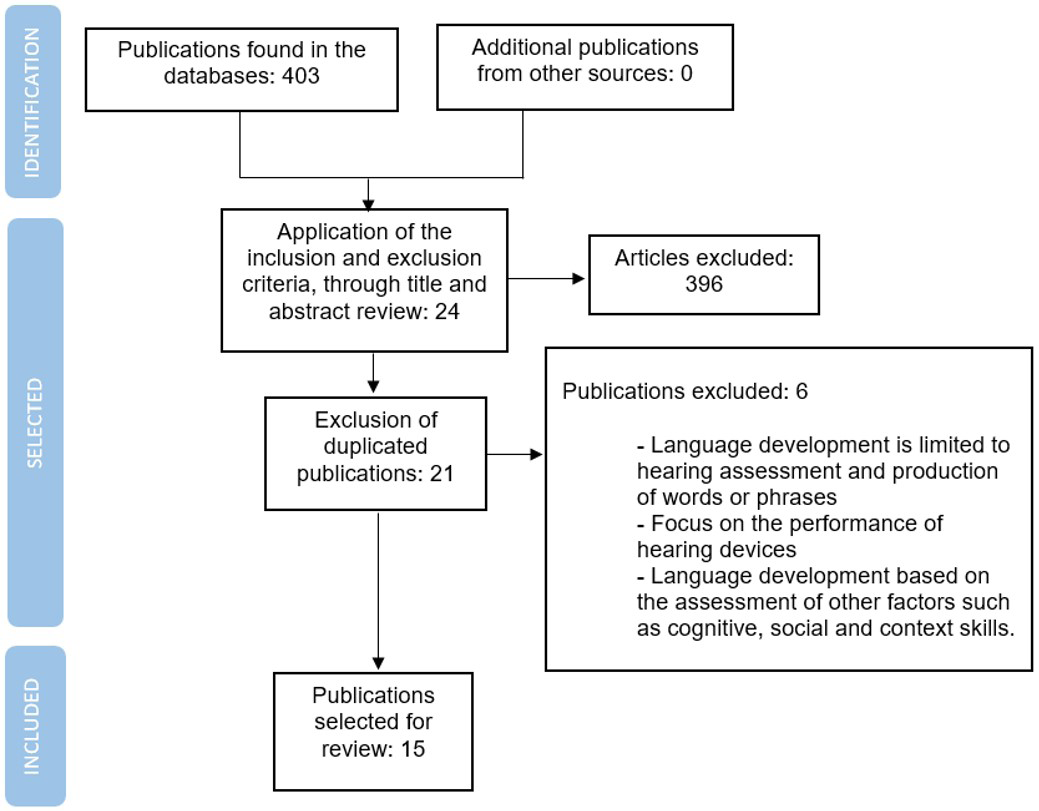
PRISMA flowchart of the body of evidence

A structured review of the scientific literature was carried out, consisting of the search, selection, analysis, and synthesis of the information based on the PICO question, namely - P: prelingually deaf children; I: assessment of the language development during their first six years of life, according to the MeSH terms (deafness, hearing loss, child, language development, assessment and testing) along with search filters. After consulting the ensuing databases: PubMed^(8)^, Lilacs^(9)^, Ibecs^(10)^, Trip DataBase^(11)^, Cochrane Library^(12)^, Clinical Trial^(13)^, and Nice^(14)^, were primarily included in the present review the following types of studies: systematic reviews, health technology assessments, randomized clinical trials, in addition to cohorts, case-control studies and further related researches conducted in the past 5 years. The recommendations of The Preferred Reporting Items for Systematic Reviews and Meta-Analyses (PRISMA) have been followed, and its flowchart, as presented in Figure 1, was applied to summarize the present selection of articles. The researchers selected and analyzed each publication that met the following inclusion criteria: having a population of prelingually deaf children, evaluating any aspect of language development, using treatments that involve cochlear implants, hearing aids, speech-language therapy, or none, at any language. The studies that included full text or abstract with related results were prioritized. The exclusion criteria were: only evaluating hearing or speech production, referring to central, sudden, transient or psychological deafness, deaf-blindness, or further disabilities caused by infections with other neurological consequences or concerning autism spectrum disorders.

## DATA ANALYSIS

An exhaustive reading and data collection of the selected articles was carried out, and the information was compiled in charts using Microsoft Excel. The following data was plotted: author and year, title, population and age, type of deafness and intervention, language assessments performed and related outcomes. Such information was grouped into the following language assessment variables: general language development, receptive language, expressive language, other language elements, and additionally, sign language.

After this process, evidence charts were prepared with the Grade Pro GDT program, and the GRADE methodology was followed to analyze the information as well as to consider the importance of the outcomes (Chart 1): general language development (critical - 7), receptive language development (critical - 7), and expressive language development (critical - 7). Based on the selected body of evidence, an additional outcome was attached, concerning further language elements such as morphology, syntax, semantics, number of words, length of sentences and conversations, and pragmatics (important - 5)^(15)^.

**Chart 1:** Outcomes proposed by the PICO question and the studies review

**No.**	**Outcome**	**Qualification**	**Description**	**Conclusions**
1	General Language Development (LD)	7	Critical	Its delay or advance determines the development in other areas
2	Receptive Language (RL)	7	Critical	Its delay or advance determines the development in other areas
3	Expressive Language (EL)	7	Critical	Its delay or advance determines the development in other areas
4	Other linguistic elements (O)	5	Important	Attached after the review of the selected publications

## RESULTS

The research process for related and relevant articles is detailed in Annex A. Initially, we analyzed 403 publications, which were reduced to 21 after applying the inclusion and exclusion criteria, along with the elimination of duplicates. Subsequently, were excluded the studies that did not correspond to the established objective due to: 1) defining language development as deriving from the listening ability, 2) focusing on the functioning of hearing devices, and 3) inferring the development of language from the assessment of other factors such as cognitive, social and cultural context skills. Finally, 15 publications were considered for the present review, with the corresponding data described in Annex B. The results related to the language development assessment are detailed in the following sections, according to the importance of selected outcomes.

### Language development assessment

Overall, the findings obtained herein show that it is possible and feasible to perform language development assessments in prelingually deaf children during their first six years of life, and that the resulting data can also be used to maintain a long-term follow-up. Regarding language development, the moderate quality of evidence (Chart 2) suggests that it is reasonable to trust the assessments’ results, provided that they are accompanied by an evaluation of receptive and expressive language development (Chart 3). So far, it is possible to infer limitations related to the understanding of words, sentences or ideas, as well as in the ability to perform the mental processes required to materialize words, gestures and signs. Among the selected studies, there is no clear consensus to determine the choice of one or another assessment tool.

**Chart 2:** Quality of the evidence according to the GRADE Manual and GRADEPro GDT^(15)^ software with the question: Language development assessment of prelingually deaf children during their first 6 years to detect limitations in language development

***Certainty assessment***								***Certainty***	***Importance***
***No. of studies***	**D**	**Study design**	**Risk bias**	**Inconsistency**	**Indirect evidence**	**Imprecision**	**Other considerations**		
*4*	DL	observational studies	not serious	not serious	not serious	not serious	strong association	⨁⨁⨁◯ Moderate	CRITICAL
*7*	LR	randomized trials	not serious	not serious	not serious	not serious	none	⨁⨁⨁⨁ High	CRITICAL
*6*	LE	randomized trials	not serious	not serious	seriousa,b	not serious	none	⨁⨁⨁◯ Moderate	CRITICAL
*8*	O	observational studies	not serious	not serious	not serious	not serious	none	⨁⨁◯◯ Low	IMPORT.

a. Population not differentiated between users of cochlear implants and hearing aids.

b. Population not differentiated between deaf children with deaf and hearing families.

**Chart 3:** Organization of the selected studies by language assessment topics

Language assessment issues in the studies	Number of studies
Studies that assess only general language development	1
Studies that assess language development, receptive language and expressive language	5
Studies that assess only receptive language or only expressive language	2
Studies that assess other elements of language to justify the development of receptive or expressive language	3
Studies that assess pragmatics	2
Studies that prioritize orality	10
Studies that prioritize gestural and sign language	3
Studies that take SL into account	1

### Assessment of the receptive and expressive language development

In total, seven of the selected researchers opted for the assessment of receptive and expressive language, while only three considered other elements to justify their results. A high quality of evidence (Chart 2) for the evaluation of receptive language suggests a mostly trustworthy set of assessments’ results in this area, aimed at understanding language elements. It is considered that this level of evidence is supported by two aspects: the fact that part of the data comes from randomized clinical trials, and the fact that the results corroborate the findings of Rodríguez-Ortiz et al.^(16)^ adapting the concepts of receptive and expressive language to child signers who are users (including natives) of sign language.

Even though expressive language is an observable element of language and therefore easier to measure, a moderate quality of evidence (Chart 2), when compared to other outcomes, indicates that more biases may be experienced in the evaluation and interpretation of the results obtained from deaf children in this area. This situation is probably related to the possibility that such biases are associated with the occurrence of deaf children's repetition of words and phrases without a clear meaning^(1)^, versus a production of elements that corresponds to an actual development.

### Assessments of other language elements

As mentioned in previous sections, the elements of form and content of language are the fundamental pieces that bestow meaning to both reception and expression^(7)^, in other words, they guarantee that such certainty of reception or expression has observable linguistic elements. A low quality of evidence (Chart 2), when compared to other outcomes, imply that the evaluation of these aspects is not reliable, especially when isolated, unaccompanied by other results such as receptive or expressive language. The evaluation tools in the studies selected for this area, as attached in Annex B, arise from the traditional - oral assessment of the language, thus being feasible to apply when orally produced data is obtained. When considering the study regarding the adaptation of the tools for language assessment in signing children by Rodríguez-Ortiz et al.^(16)^, there is a noticeable demand for research and propositions of tools able to evaluate formal linguistic elements of sign language, such as cherology^(17)^.

In this regard, it is worth considering the elements of language use and pragmatics^(7)^. Despite the identification of only 2 studies that address this element (Chart 3), it is important to highlight that this type of evaluation, regardless of being included in a low level of evidence, considers other factors such as cognitive development from the Theory of Mind^(18,19)^, surpassing IQ assessment tools. The latter usually use items whose processing implies, a priori, an oralistic approach of language. However, taking into account other cognitive considerations is proven to be valuable for the construction of new types of language development assessments.

### Oral language versus sign language

The two systematic reviews included herein^(5,20)^, although presenting different conclusions and prioritizing two different interventions, highlight two pivotal factors: the cost and resources related to the implants, along with their questionable effectiveness in the long term, by failing to raise the child's language development to the apparent standard level of a hearing subject.

The studies that consider oral rehabilitation and cochlear implants do not include in their results the intervening variables related to the possible presence of gestures or even approaches to sign language. This situation is surely explained by the historical prejudices against sign language, where this language is seen as a supplementary instrument or even as a detrimental hindrance to oral skills^(20)^. The systematic review conducted by Hall ^(20)^. is the only publication found that specifies their results on an overall effective language development in cases where implants are used in tandem with sign language (result obtained from the study by Davidson et al.^(21)^). This feat can be compared with the brief note found in the publication by Meinzen-Derr et al.^(22)^, mentioning that their randomized clinical study with an oral deaf population should be replicated, in the future, with a signing deaf population.

### Further aspects to consider

Most studies agree that the age of the deafness diagnosis coupled with an early intervention can generate an improved and more timely response to better cater for the needs of deaf children; however, there is no consensus on how to choose the information type, prioritizing oral rehabilitation and leaving sign language aside.

The majority of the studies focus on a level of language development after the cochlear implantation, close or similar to the hearing child, but never the same; except for the researches that consider sign language and gestures, placing the deaf child on the same level as the hearing person. Therefore, the possibilities of sign language should be taken into account when talking about development and assessment, even when it is acquired or learned by hearing children without hearing loss^(16)^. Such considerations challenge other reviews and studies that do not contemplate costs and resources in their outcomes, as well as the values and preferences of both mothers and fathers with deaf children.

## DISCUSSION OF THE RESULTS

The published literature regarding the efficacy of interventions and the assessment of the language of prelingually deaf children seems to be reliable, as long as based on the lack or reduction of hearing and the need for oral speech^(23)^. Nevertheless, when the problem is approached from an integrated perspective of language, the evidence is still scarce and there is no consensus for determining the best way to perform the evaluations. Undoubtedly, this issue is connected to a problem that precedes the assessments: the intervention options, or rather, the decisions made by medical professionals who mostly advocate for the prompt rehabilitation of deaf children into normative hearing standards. From this starting point, language assessment becomes biased towards the observable elements that the deaf child can orally produce, from which the presence of language is inferred. However, this approach overlooks the possibility that the child has achieved some language with elements that surpass the sound production. The proposals made by Paul Watzlawick et al.^(24)^ provide a spectacular paradigm shift in human interaction, where this “beyond the production of language” is put in context as the first axiom of communication: “it is impossible not to communicate”, which perfectly describes the fact that even if a child does not produce speech sounds, there are other instances of language working in their head.

It is crucial to perform an early detection and intervention of deafness, and there is sufficient evidence to consider it as the best way to prevent all sort of problems for deaf children, not only language issues. With that in mind, and considering the aforementioned, it is time to start instigating some extreme paradigm shifts in order to allow language to be addressed in an integrated approach and in all its possible modalities. The present review considers to be extremely important the valuable contribution that the participation of deaf, speaking, and signing professionals, both medical and from other areas, could have in the production of scientific evidence that take into consideration the patient’s values and preferences. This approach would certainly make it possible to narrow the gaps between the lack of consensus and the communication barriers. For this purpose, not only research is needed, but also an epistemological review of the treatments and results historically directed towards oralization.

The electronic assistive devices for the hearing impaired, such as cochlear implants and hearing aids, deserve special mention. The present study has no intention whatsoever to dismiss, deny, or prohibit its use, on the contrary, the interest herein is directed to reconsider how these devices are offered and provided to mothers, fathers and deaf children. The burgeoning growth of technology in recent years cannot be underestimated, and it would be unfathomable not to acknowledge that the scope and benefits of such implants will surely show an improvement. However, faced with this accelerated evolution, it is better to be sure of all intervening factors. On the one hand, it is opportune to continue building evidence on the economic impact of cochlear implants for families^(5)^. On the other hand, is pertinent to begin to describe the long-term adverse effects in an integrated approach, as already stated, for example, the occurrence of otosclerosis or residual hearing loss^(25)^.

Considering the course of the discussion thus far, it is important to mention the urgency of transmitting this knowledge to all of the audiologists who determine the fate of deaf children. To take into account the values and preferences of these patients is not only a medical issue, but a social and cultural matter that is directly linked to the struggle that the Deaf Community deals with worldwide. Therefore, it is necessary to ensure a complete and continuous training concerning hearing loss, deafness, the deaf community, deaf culture and sign language, to establish this long overdue integrated approach. Along with these changes, an impact is expected not only on the interventions, but on the entire regulatory apparatus which is also responsible for the fate of the deaf child.

Additionally, it is worth mentioning that the clinical studies may be affected by an imprecision bias due to their relatively small samples, but they do have good designs and methodologies. It must be reckoned that the diversity of deafness is too extensive, leading these studies to strive to obtain more homogeneous samples. Even so, it is noticeable that there is a lack of differentiation between populations that use implants or hearing aids, or those who have a deaf or hearing family, points that tend to diminish the quality of the evidence related to expressive language. The present review must concede that it may be affected by an indirect evidence bias, given that the selected studies assess various degrees of hearing loss, from mild to severe, when it is acknowledged that language development will differ according to each case. Furthermore, reports on the use of cochlear implants and hearing aids should be considered as extra feedback, since they are not part of the research question. Nevertheless, we expect that they inspire future research and systematic reviews.

## CONCLUSIONS

Based on the quality ratings of the body of evidence regarding the evaluation of the development of prelingually deaf children, it is considered that there is sufficient reliable information to perform assessments during the first six years of the children’s life. However, the interpretation of the results must consider that the obtained data on general language development should be mostly supported by the evaluation of receptive language over expressive language, moreover, the evidence can be enriched by including assessments of the formal linguistic elements of the oral modality, such as gestures, along with the pragmatic components of the communication processes.

Further research is required for the assessment of formal elements of the gestural modality of communication, such as the sign languages, which enable the construction of tools (inventories, scales, observation protocols) that allows to compare the results of the development of deaf children according to the intervention they have been exposed to, or the choice they have made.

## Annex A. Research strategy and expressions used in the main sources of information

**Table:**

**RESEARCH PERFORMED. Search date: 02-08-2022**					
**No.**	**PLATFORM**	**RESEARCH EXPRESSION**	**FILTERS**	**RESULTS**	**RESULTS AFTER THE INCLUSION AND EXCLUSION CRITERIA**
1	PubMed	**(Child AND (Deafness OR Hearing Loss)) AND Language Development AND (Language Evaluation OR Evaluation OR Test)**	Child: birth-18 years old	293	18
			Newborn: birth-1 month old		
			Baby: birth-23 months old		
			Baby: 1-23 months old		
			Preschool Child: 2-5 years		
			Last 5 years		
2	BVS-LILACS	**(Child AND (Deafness OR Hearing Loss)) AND Language Development AND (Language Evaluation OR Evaluation OR Test)**	Main subject: deafness, language development	11	1
			Last 5 years		
3	BVS-IBECS	**(Child AND (Deafness OR Hearing Loss)) AND Language Development AND (Language Evaluation OR Evaluation OR Test)**	Main subject: deafness, language development	3	0
			Last 5 years		
4	Clinical Trial	**(Child AND (Deafness OR Hearing Loss)) AND Language Development AND (Language Evaluation OR Evaluation OR Test)**	Age: 6 years	28	0
5	Trip Medical DataBase	**P(Child, Deafness) I (Language Development)**	Since 2017	11	2
6	Cochrane Library	**Deafness AND Child AND Language evaluation**	-	37	2
7	National Institute for Health and Care Excellence NICE	**Deafness**	-	20	1
**TOTAL**				**403**	**24**
**REMOVAL OF DUPLICATES**					**21**
**SECOND SELECTION OF ARTICLES FOR REVIEW (** **FIGURE 1** **.)**					**15**

**Table:** Annex B. Studies selected for the review

No.	Author	Title	Patients’ age	Type of deafness and interventions	Related language assessment	Related results	O^1^
Systematic Reviews and Evaluations of Health Technology SR/ETS							
1	Ontario Health Quality^(5)^	Implantable Devices for Single-Sided Deafness and Conductive or Mixed Hearing Loss: A Health Technology Assessment	#NR^2^	Unilateral, conductive and mixed deafness	Speech Intelligibility Rating (SIR)	Improvement in language development.	LD^3^
			Adults	cochlear implant		High cost and resources in the process after cochlear implantation.	
			Children				
2	Hall^(20)^	What You Don't Know Can Hurt You: The Risk of Language Deprivation by Impairing Sign Language Development in Deaf Children	#NR	Deafness in the early developmental stages	Introduction of sign language, oral language or both for the language development of deaf children.	Limited evidence on the efficacy of sign language as a first language.	O^4^
			Deaf children in general			Medical intervention classifying deaf children as “hard of hearing”. Oralism as a form of prevention the exposure to sign language.	
						Delayed speech affecting the development of brain structures.	
						The cochlear implant does not guarantee language development at two years old. But it is guaranteed in children who sign from birth.	
						Language deprivation appears to be the cause of deaf people's poor language outcomes.	
RCTs Randomized Clinical Trials							
3	Meinzen-Derr et al.^(22)^	A Technology-Assisted Language Intervention for Children Who Are Deaf or Hard of Hearing: A Randomized Clinical Trial	Intervention: 21 deaf children from 3 to 12 years old.	Mild to severe bilateral hearing loss	Long phrases when communicating	Increase in TALI vs. TAU scores:	RL^5^
			Control: 20 deaf children	Implant and hearing aids	MLU Syntax	MLU (β = 0.91 against 0.15, *P* < 0.0001)	EL^6^
				Intervention:	NWD semantics	NWD (β = 1.21 against 0.26, P = 0.005)	O
				TALI: children with technology-assisted language intervention	MTL speech	MTL (β = 11.04 against 2.65, *P* = 0.007)	
				Control:	Clinical assessment of language fundamentals	Increase in the mean score	
				TAU: children with usual treatments	Receptive language scores	Receptive:	
					Expressive language scores	TALI: 80.0 to 90.6 (p=0.008)	
						TAU: 82.1 to 83.6 (p=0.09)	
						Expressive.	
						TALI: 77.6 to 86.1 (p=0.01)	
						TAU: 77.5 to 79.9 (p= 0.21)	
4	Monshizadeh et al.^(26)^	The effectiveness of a specifically-designed language intervention protocol on the cochlear implanted 2. Children's communication development	Intervention (I): 26 children from 20 to 24 months	Cochlear implant CI users,	Bayley Scales of Infant and Toddler Development - Third Edition.	Mean score (standard deviation),	RL
			Control (C): 25 children	Intervention: auditory verbal rehabilitation and cognition-based intervention	General Development GD	GD:	EL
				Control: auditory-verbal rehabilitation.	Receptive communication RE	I: 91 (10.73)	
					Expressive communication EX	C:73.64 (8.7)	
						Correlation of both groups: t=6.33 df=49, (p=0.001)	
						RE: Correlation of both groups (p=0. 001)	
						EX: Correlation of both groups (p=0. 01)	
Cohort Studies							
5	Yoshinaga-Itano et al.^(18)^	Early Intervention, Parent Talk, and Pragmatic Language in Children With Hearing Loss	124 children from 4 to 7 years old	Bilateral hearing loss, mild to severe	Pragmatic verification checklist.	Mean scores (standard deviation), P value, and confidence interval.	O
					Pragmatic prediction by chronological age CA	CA 0.15 (0.01) p<0.01 (7.73 - 8.90)	
					Pragmatic Prediction by IQ IQ	IQ 0.08 (0.01) p<0.01 (0.05 - 0.11)	
					Prediction of pragmatics by the education of mothers EM	EM 0.23 (0.10) p<0.17 (0.04 - 0.42)	
					Pragmatic prediction by the number of parental words per minute PP	PP 0.04 (0.01) p<0.003 (0.02 - 0.07)	
					Pragmatic Prediction by the Degree of Loss DL	DL -1.08 (0.50) p<0.34 (-2.07 - 0.08)	
					Pragmatic prediction by complying with EHDI	EHDI 1.00 (0.49) p<0.043 (0.03 - 1.97)	
6	Li et al.^(27)^	Developmental performance among pediatric candidates for cochlear implantation	500 children from 6 to 72 months	Severe profound hearing loss	Gesell development skills	General development delay (p< 0.001)	LD
				CI Cochlear Implant Candidates	Overall development	Delay in verbal and non-verbal skills (p<0.05)	
					Language development	Worst language development (normal rate 4.2%)	
Case-control studies							
7	Guo and Spencer^(28)^	Development of Grammatical Accuracy in English-Speaking Children With Cochlear Implants: A Longitudinal Study	10 deaf children from 3 to 5 years old	Prelingual deafness	Grammar Communication Units (PGCU or C Units) in storytelling.	Auditory experience duration	O
			Cochlear Implant (CI)	CI Cochlear Implant	Significant effect of the listening experience duration on PGCU	F(2, 18) = 3.99, p = .037, η _p_^2^ = .31	
			Control group: 10 children with normal hearing from 3 to 5 years old.		PGCU production in children with CI is lower than in children with normal hearing at 3 years of implantation	PGCU Production:	
					PGCU production in children with CI is lower than in children with normal hearing at 4 years of implantation	At 3 años:	
					PGCU production in children with CI is lower than in children with normal hearing at 5 years of implantation	F(1, 18) = 1. 75, **p = . 20**, η _p_^2^ = 0.09	
						At 4 years:	
						F(1, 18) = 4.78, p = .04, η _p_^2^ = .21	
						At 5 years:	
						F(1, 18) = 5.74, p = .03, η_p_^2^ = 0.24	
8	Wie et al.^(29)^	Long-Term Language Development in Children With Early Simultaneous Bilateral Cochlear Implants	19 children from 5 to 18 months	Congenital profound deafness	Language skills in 10 moments	Children with CI compared to normal hearing. Significant differences according to the months after implantation.	LD
			Control group: 19 children with normal hearing	Bilateral CI Cochlear Implants	Understanding-Conceptual Subscale of the Minnesota Child Development Inventory (MCDI) Parent Questionnaire	DGL:	RL
					General development of the DGL language	Before 12: no significant differences.	EL
					Mullen Scale of Early Learning (MSEL)	12 months: p = 0.004; d = 1.21	
					RE receptive general language	From 36 months: no significant differences	
					Expressive General Language EX	RE:	
					British Pictorial Vocabulary Scale, Second Edition (BPVS II)	3 months: p < 0.001; d = 2.56	
					VRE Receptive Vocabulary	36 months: no significant differences	
					Wechsler Preschool and the Primary Scale of Intelligence, Third Edition (WPPSI-III) Picture Naming Subtest	EX:	
					VEX Expressive Vocabulary	3 months: p < 0.001; d = 2.51	
						48 months: no significant differences.	
						VRE:	
						36 months: no significant differences	
						48 months: p = 0.02; d = 0.88	
						60 months: p < 0.001; d = 1.53	
						72 months: p < 0.001; d = 1.36	
						VEX:	
						No significant evidence	
9	Werfel^(30)^	Morphosyntax Production of Preschool Children With Hearing Loss: An Evaluation of the Extended Optional Infinitive and Surface Accounts	18 pre-school children with 45 to 6 months	Moderate to severe deafness	Hadley protocol.	X2 test: spontaneous language does not differ from the type of amplification (p>0.05)	O
			Control group, normal hearing	Cochlear implants and hearing aids	Brown's morphemes.	Mean scores in three groups: deaf - control 1 - control 2:	
			1. Same age (18)		Expressive Language Subscale - Test of Early Language Development-Third Edition (TELD-3)	MLU: 4.37 - 5.82 - 5.22 (p=0.25)	
			2. Same language (18)		Morphemes, MLU phrase length	NDW: 146.44 - 183.50 - 173.33 (p=0.065)	
					Semantics, number of different words NDW	TNW: 413.22 - 525.89 - 497.72 (p=0.174)	
					Total number of words TNW		
10	Netten et al.^(19)^	Can You Hear What I Think? Theory of Mind in Young Children With Moderate Hearing Loss	44 deaf children from 3 to 5 years old	Hearing loss, preferred to use spoken language	Reynell Developmental	Deaf children correlations (Partial r)	RL
			Control group: 101 hearing children		Language Scale, Schlichting	Of children, ToM and RE:	EL
					Expressive Language Test and Scales for Parents	Similar desire: 0.36 (p<0.05)	
					Obtained from the child N:	Dissimilar desire: 0.24 (p>0.05)	
					Correlation of RE receptive language and EX expressive language with aspects of theory of mind ToM, compared to the control group.	False belief: 0.56 (p<0.001)	
					Obtained from parents P:	Of children, ToM and EX:	
					Correlation of CL language comprehension and EL language expression with aspects of the theory of mind, compared to the control group.	Similar desire: 0.32 (p<0.05)	
						Dissimilar desire: -0.01	
						False belief: 0.35 (p<0.05)	
						From parents ToM and CL:	
						Similar desire: 0.09 (significant difference)	
						Dissimilar desire: 0.26 (p<0.01)	
						False belief: 0.24 (p<0.01)	
						From ToM and EL parents:	
						Similar desire: 0.22 (p>0.05)	
						Dissimilar desire: 0.13 (p>0.05)	
						False belief: 0.29 (p<0.001)	
11	Aslıer et al.^(31)^	The influence of age and language on the developmental trajectory of the theory of mind in children with cochlear implants	111 children from 36 to 132 months	Congenital bilateral profound deafness	Sally-Ann (Theory of Mind ToM test)	Higher language scores are associated with better performance on the Sally-Ann test (p<0.05)	O
			Control group: more than 99 children	CI Cochlear Implant	Peabody picture vocabulary		RL
					Receptive language		
Observational Studies							
12	Scarabello et al.^(32)^	Language evaluation in children with pre-lingual hearing loss and cochlear implant.	30 children from 36 to 72 months	Severe and profound bilateral sensorineural hearing loss.	ABFW Child Language Test-Part B - Vocabulary. expressive oral language.	Correlation of factors with ABFW scores: significant differences and p-values for each test item. Refer to the article.	O
				CI Cochlear Implant	Peabody Picture Test. Receptive oral language	Correlation of factors with Peabody test scores:	
						Surgery age: −0.297 (p=0.168)	
						Assessment age: −0.645 (p= 0.001)	
						Implant use time: −0.332 (p= 0.122)	
						Phonemes: 0.020 (p= 0.929)	
						Words: 0.012 (p= 0.957)	
13	Kutlu et al.^(33)^	A study on the association of functional hearing behaviors with semantics, morphology, and syntax in cochlear-implanted preschool children	48 children from 3 to 5 years with 11 months.	Severe and profound bilateral hearing loss	Test of Early Language Development - Third Edition (TELD-3)	Relationship between semantics, morphology, syntax, and functional listening ability (p<0.05)	LD
				CI Cochlear Implant	Receptive language	Correlation between receptive language, semantic receptive language, and grammatical receptive language with verbal communication: (r = 0.781; r = 0.729; r = 0.787) (0.627; p < 0.01)	RL
					Expressive language	Correlation between expressive language, semantic expressive language and grammatical expressive language with verbal communication	EL
					Functioning Form after Pediatric Cochlear Implantation (FAPCI)	(r =0.797; r = 0.749; 0.782) (0.757; p < 0.01)	
Other studies and publications							
14	Rodríguez-Ortiz et al.^(16)^	A Spanish Sign Language (LSE) Adaptation of the Communicative Development Inventories	55 children from 8 to 36 months	Deaf signers	Adaptation of the MacArthur Communicative Development Inventory (CDI) Fenson *et al*., 1993.	They understand more signs than they produce. Lowest and highest age:	RL
					Comprehension of signs compared to those produced	At 8-11 months, z(16) = 3.52, p < .001, η 2 = 12.686;	EL
					Receptive and expressive language	At 32-36 months, z(31) = 4.83, p < 0.001, η 2 = 12.309.	
						Receptive and expressive language development is similar	
						At 8-11 months, r(16) = 0.64, p = 0.008.	
						At 32-36 months, r(31) = 0.81, p < 0.001.	
15	Goldin-Meadow^(34)^	Using Gesture To Identify and Address Early Concerns About Language and Pragmatics	Deaf children in general	Children who use gestures before words or signs	Observation of gestures (non-standardized - signs) to detect developmental and pragmatic problems.	NR	O
						Suggests possible effectiveness	

1 D: outcomes included

2 NR: not reported data

3 LD: Publication included in the outcome General language development

4 O: Publication included in the outcome Other language elements

5 RL: Publication included in the outcome Receptive Language

6 EL: Publication included in the outcome Expressive Language
