# Supplementary material for: Is speech enough? Language development evaluation in the first 6 years of prelingually deaf children. A systematic review
Source: Codas. 2023 Nov 10;35(5):e20220084. doi: 10.1590/2317-1782/20232022084en (PMC10688294; doi:10.1590/2317-1782/20232022084en)
Supplement: Translation

# Translation: ¿Basta con el habla? Evaluación del desarrollo del lenguaje en niños sordos prelocutivos durante los primeros seis años de vida. Una revisión sistemática

**DOI:** 10.1590/2317-1782/20232022084es

**Authors:** Raúl Francisco Lara Barba, Yadira Niyireth Angamarca Guanuche, Andrea Lorena Mera Herrera, Erick Fabricio Gudiño Chinchin, Victor Hugo Altamirano Sampedro, María Belén Mena Ayala

**Affiliations:** 1 Facultad de Ciencias de la Discapacidad, Atención pre-hospitalaria y Desastres, Universidad Central del Ecuador ­- UCE - Quito (Pichincha), Ecuador.; 2 Universidad Central del Ecuador - UCE - Quito (Pichincha), Ecuador.

**Keywords:** es, Pérdida Auditiva, Lenguaje, Lenguaje Infantil, Evaluación de la Discapacidad, es, Pérdida Auditiva, Lenguaje, Lenguaje Infantil, Evaluación de la Discapacidad, es, Pérdida Auditiva, Lenguaje, Lenguaje Infantil, Evaluación de la Discapacidad, es, Pérdida Auditiva, Lenguaje, Lenguaje Infantil, Evaluación de la Discapacidad, es, Pérdida Auditiva, Lenguaje, Lenguaje Infantil, Evaluación de la Discapacidad

## Abstract

**Objetivo:**

Sintetizar la información científica relevante sobre la evaluación del desarrollo del lenguaje en niños sordos prelocutivos durante los primeros seis años de vida, con el fin de determinar si es suficientes para confirmar la presencia de algún desarrollo de lenguaje en esta población, o si se requiere un enfoque más integral.

**Estrategia de investigación:**

Se realizó una revisión estructurada de la literatura científica en las siguientes bases de datos: PubMed, Lilacs, Ibecs, Trip DataBase, Cochrane library, Clinical Trial y Nice.

**Criterios de selección:**

revisiones sistemáticas, evaluaciones de tecnologías sanitarias, ensayos clínicos aleatorizados, estudios observacionales de cohorte y casos - controles; incluyendo publicaciones sobre evaluación de cualquier aspecto del desarrollo de lenguaje, cualquier intervención y en cualquier idioma, se excluyeron si únicamente evalúan audición o habla, sordera central, súbita, transitoria, sordoceguera, con otras discapacidades o con trastornos del espectro autista.

**Análisis de datos:**

se usó la metodología GRADE para analizar la calidad de la evidencia.

**Resultados:**

es factible realizar evaluaciones del desarrollo de lenguaje a niños sordos prelocutivos. Se obtiene una calidad de la evidencia moderada que sugiere cierta confianza en los resultados de las evaluaciones, siempre y cuando vengan acompañadas de una apreciación integral de otros elementos lingüísticos.

**Conclusión:**

los resultados de las evaluaciones de lenguaje deben sostenerse, en mayor medida, en datos sobre el lenguaje receptivo y expresivo, y que cuyas evidencias pueden enriquecerse al adjuntar evaluaciones de elementos lingüísticos formales tanto de la modalidad oral como gestual, y de los componentes pragmáticos de los procesos comunicacionales.

## INTRODUCCIÓN

El abordaje de la sordera prelocutiva en niños ha sido, desde múltiples perspectivas, una cuestión difícil de considerar en vista de que la mayor parte de sus planteamientos surgen de una visión médica - rehabilitadora, y además oralista del componente físico de la sordera: la deficiencia auditiva^(1)^. Es decir, frente a una enfermedad o condición de discapacidad de la audición, se busca, a toda costa, recuperarla de tal forma que el niño sea capaz de “hablar”; quienes se adhieren a esta idea parten de la hipótesis de que basta con la audición para desarrollar integralmente el lenguaje^(2)^. Por tales razones, se hace necesario considerar si la evaluación de la audición y de la producción del habla están considerándose suficientes para establecer algún desarrollo de lenguaje en niños sordos o si es prudente optar por un enfoque más integral^(3)^.

Los seis primeros años de vida de todo niño, sordo u oyente, son cruciales en la determinación de su desarrollo posterior, incluido el lenguaje; mientras que durante los tres primeros años suceden los fenómenos de adquisición natural del lenguaje, a los tres posteriores se garantiza el afianzamiento del mismo, para que a partir de los seis tengan lugar procesos de aprendizaje organizado y planificado del lenguaje^(4)^, sin embargo, tales descripciones no siempre son aplicables a aquellas situaciones donde el lenguaje podría no apegarse a la modalidad oral. Por lo tanto, enfrentar una falta de seguimiento, implica riesgos importantes en el desarrollo del lenguaje que podrían prevenirse procurando el accionar de profesionales especializados en esta población^(3)^. La presente revisión dirige su atención a la población de niños sordos prelocutivos cuya condición responde a múltiples causas, así como consecuencias, entre las que están: un impacto negativo sustancial en el sistema auditivo en desarrollo y en el desarrollo del lenguaje, el riesgo de retraso del habla (problemas para producir sonidos y/o comprender el habla), rendimiento académico deficiente, problemas de comportamiento y disminución de la calidad de vida^(5)^.

Cuando la producción de palabras y el habla no son suficientes para predecir algún desarrollo de lenguaje, necesariamente hay que recurrir a otras opciones. En los años setenta, varias corrientes filosóficas, lingüísticas y sociológicas impulsaron movimientos que sostenían el entendimiento amplio del lenguaje desde la funcionalidad y la pragmática, es decir, había que interpretar el lenguaje infantil desde la comunicación; desde la intención y la situación en la que el niño capta mensajes^(6)^. Los niños sordos no están fuera de estas aproximaciones; sin embargo, les acaece un factor extra; un mundo construido para oyentes. Si su desarrollo se dirige a la esfera oral, su proceso de adquisición no es espontáneo y natural, sino un difícil aprendizaje planificado por el adulto; si su desarrollo es gestual, suele ser natural, pero con limitaciones en relación en el entorno en el que nacen^(4)^. Una evaluación completa del lenguaje se considera una tarea compleja de realizar, en vista de agrupar varios componentes y de diferir en su conceptualización según la perspectiva que se tome. De manera general el lenguaje puede agrupar elementos de producción y comprensión, donde intervienen elementos de audición, de fonación, de forma (fonología y morfología), de contenido (semántica) y de uso (pragmática)^(7)^.

La concepción del lenguaje y del desarrollo de lenguaje es, evidentemente, amplia y sobrepasa el hecho de la audición y producción de palabras. Sin embargo, un vistazo a la realidad actual, parece seguir empujando el destino de los niños sordos hacia la audición aparentemente “normal”, sin considerar otras opciones. Por tal motivo, la presente revisión sistemática busca responder a la pregunta de investigación: ¿Qué evaluaciones del desarrollo del lenguaje se están realizando en la actualidad a los niños sordos prelocutivos durante sus primeros 6 años de vida?

## OBJETIVO

Identificar, seleccionar, evaluar y sintetizar la evidencia relevante disponible sobre la evaluación actual del desarrollo del lenguaje durante los primeros seis años de vida de niños sordos prelocutivos.

## ESTRATEGIA DE INVESTIGACIÓN

Se realizó una revisión estructurada de la literatura científica, consistente en búsqueda, selección, análisis y síntesis de la información a partir de la pregunta PICO; P: niños sordos prelocutivos I: evaluación del desarrollo de lenguaje durante los primeros seis años de vida, con términos MeSH (deafness, hearing loss, child, language development, evaluation, test) y filtros de búsqueda. Luego de consultar las siguientes bases de datos: PubMed^(8)^, Lilacs^(9)^, Ibecs^(10)^, Trip DataBase^(11)^, Cochrane library^(12)^, Clinical Trial^(13)^ y Nice^(14)^, se incluyeron en la revisión primordialmente estudios de tipo: revisiones sistemáticas, evaluaciones de tecnologías sanitarias, ensayos clínicos aleatorizados, y además se consideraron: estudios de cohorte, de casos y controles y otros relacionados, de los últimos 5 años. Se ha seguido las recomendaciones de The Preferred Reporting Items for Systematic Reviews and Meta-Analyses (PRISMA), cuyo diagrama de flujo, que se presenta en la Figura 1, resume la selección de artículos realizada. Los investigadores seleccionaron y analizaron cada publicación que cumplió con los criterios de inclusión: que tengan una población de niños sordos prelocutivos, que evalúen cualquier aspecto del desarrollo de lenguaje, y cuyo tratamiento incluye implante coclear, audífono, terapia de lenguaje o ninguno, en cualquier idioma y se priorizaron aquellos que incluían texto completo o abstract con resultados relacionados; y de exclusión: aquellos que únicamente evalúan audición o producción de habla, sordera de tipo central, súbita, transitoria, psicológica, sordoceguera, o con otras discapacidades adicionales, provocadas por infecciones con otras consecuencias neurológicas o en relación a trastornos del espectro autista.

**Figura 1:** Diagrama de flujo PRISMA sobre la conformación del cuerpo de la evidencia

## ANÁLISIS DE LOS DATOS

Se procedió a realizar una lectura y recolección de datos exhaustiva de los artículos seleccionados, cuya información se recopiló en cuadros, por medio de Microsoft Excel. Se identificó la siguiente información: autor y año, título, población y edad, tipo de sordera e intervenciones, evaluaciones de lenguaje realizadas y resultados relacionados. Tal información se agrupó en las siguientes variables de evaluación del lenguaje: desarrollo general de lenguaje, lenguaje receptivo, lenguaje expresivo, otros elementos de lenguaje y, adicionalmente, lengua de señas.

Posterior a este proceso, se elaboraron cuadros de evidencia con el programa Grade Pro GDT y se siguió la metodología GRADE para analizar la información, y considerar la importancia (Cuadro 1.) de los desenlaces: el desarrollo general del lenguaje (crítico - 7), el desarrollo de lenguaje receptivo (crítico - 7), y el desarrollo del lenguaje expresivo (crítico - 7). A partir del cuerpo de la evidencia seleccionado, se adjunta un desenlace adicional en relación a otros elementos del lenguaje como: morfología, sintaxis, semántica, cantidad de palabras, longitud de frases y conversaciones, pragmática (importante - 5)^(15)^.

**Cuadro 1:** Desenlaces propuestos a partir de la pregunta PICO y de la revisión de estudios

**N°**	**Desenlace**	**Calificación**	**Descripción**	**Juicios**
1	Desarrollo general del lenguaje (DL)	7	Crítico	Su retraso o avance determina el desarrollo en otras áreas
2	Lenguaje receptivo (LR)	7	Crítico	Su retraso o avance determina el desarrollo en otras áreas
3	Lenguaje expresivo (LE)	7	Crítico	Su retraso o avance determina el desarrollo en otras áreas
4	Otros elementos del lenguaje (O)	5	Importante	Se adjunta luego de la revisión de publicaciones seleccionadas

## RESULTADOS

El proceso de búsqueda de artículos relacionados y relevantes se detalla en el Anexo A. Se obtuvieron inicialmente 403 publicaciones, que se redujeron a 21 luego de aplicar criterios de inclusión, exclusión y eliminación de duplicados. Luego, se procedió excluir los estudios que no correspondían al objetivo planteado debido a: 1) definir el desarrollo del lenguaje a partir de la habilidad auditiva, 2) mayor atención al funcionamiento de dispositivos auditivos y 3) inferir el desarrollo del lenguaje partir de la evaluación de otros factores como las habilidades cognitivas, sociales, y del contexto cultural. Finalmente se obtuvieron 15 publicaciones que se consideraron para la revisión, cuyos datos se describen en el Anexo B. Los resultados relacionados con la evaluación del desarrollo del lenguaje son descritos en los siguientes apartados, según la importancia de los desenlaces seleccionados.

### Evaluación del desarrollo del lenguaje

De manera general, los hallazgos obtenidos demuestran que es posible y factible realizar evaluaciones del desarrollo de lenguaje a niños sordos prelocutivos durante sus primeros seis años de vida, y que además los datos obtenidos sirven para mantener un seguimiento a largo plazo. En cuanto al desarrollo del lenguaje, una calidad de la evidencia moderada (Cuadro 2.) sugiere que es factible confiar en los resultados de las evaluaciones, siempre y cuando vengan acompañadas de una apreciación del desarrollo de lenguaje receptivo y expresivo (Cuadro 3.), hasta el momento se asegura que es posible inferir limitaciones en la comprensión de palabras, oraciones o ideas y en la habilidad de realizar procesos mentales para materializar palabras gestos y señales. Entre los estudios seleccionados no hay un consenso claro para determinar la elección de una u otra herramienta de evaluación.

**Cuadro 2:** Calidad de la evidencia según Manual GRADE Y software GRADEPro GDT^(15)^ con la pregunta: Evaluación del desarrollo del lenguaje durante los 6 primeros años para detectar limitaciones en el desarrollo del lenguaje en niños sordos prelocutivos

***Evaluación de certeza***								***Certeza***	***Importancia***
***Nº de estd.***	**D**	**Diseño de estudio**	**Riesgo sesgo**	**Inconsistencia**	**Evidencia indirecta**	**Imprecisión**	**Otras consideraciones**		
*4*	DL	estudios observacionales	no es serio	no es serio	no es serio	no es serio	fuerte asociación	⨁⨁⨁◯ Moderado	CRÍTICO
*7*	LR	ensayos aleatorios	no es serio	no es serio	no es serio	no es serio	ninguno	⨁⨁⨁⨁ Alta	CRÍTICO
*6*	LE	ensayos aleatorios	no es serio	no es serio	serioa,b	no es serio	ninguno	⨁⨁⨁◯ Moderado	CRÍTICO
*8*	O	estudios observacionales	no es serio	no es serio	no es serio	no es serio	ninguno	⨁⨁◯◯ Baja	IMPORT.

a. Población no diferenciada entre usuarios de implantes cocleares y audífonos.

b. Población no diferenciada entre sordos de familias de sordos y oyentes.

**Cuadro 3:** Organización de estudios seleccionados por temas de evaluación del lenguaje

Temas de evaluación del lenguaje en los estudios	Número de estudios
Estudios que evalúan sólo el desarrollo general del lenguaje	1
Estudios que evalúan el desarrollo del lenguaje, el lenguaje receptivo y el lenguaje expresivo	5
Estudios que evalúan sólo el lenguaje receptivo o sólo el lenguaje expresivo	2
Estudios que evalúan otros elementos de lenguaje para justificar el desarrollo del lenguaje receptivo o expresivo	3
Estudios que evalúan pragmática	2
Estudios que priorizan lo oral	10
Estudios que priorizan lo gestual y la lengua de señas	3
Estudios que toman en cuenta LS	1

### Evaluación del desarrollo del lenguaje receptivo y expresivo

En total, siete de los estudios seleccionados optan por la evaluación del lenguaje receptivo y expresivo, mientras que solo tres toman en cuenta otros elementos que justifiquen sus resultados. Una calidad de evidencia alta (Cuadro 2.) para la evaluación del lenguaje receptivo sugiere que se puede confiar, en mayor medida, de los resultados de evaluaciones en esta área dirigida a la comprensión de elementos de lenguaje. Se considera que tal nivel de evidencia se sostiene en dos aspectos: parte de la evidencia proviene de ensayos aleatorios clínicos y los resultados suman el aporte del estudio de Rodríguez-Ortiz et al.^(16)^, que adapta los conceptos de lenguaje receptivo y expresivo a niños signantes usuarios (incluidos nativos) de lengua de señas.

A pesar de que el lenguaje expresivo es el elemento observable del lenguaje, por ende, más sencillo de medir, una calidad de la evidencia moderada (Cuadro 2.), en comparación a otros desenlaces sugiere que pueden experimentarse más sesgos en evaluación e interpretación de los resultados obtenidos por los niños sordos en esta área. Tal situación debe relacionarse con la posibilidad de que los sesgos tengan que ver con una aceptación de la repetición de palabras y frases del niño sordo sin un sentido claro^(1)^, versus una producción de elementos que en realidad responden a un desarrollo.

### Evaluación de otros elementos del lenguaje

Como se ha mencionado en apartados anteriores, los elementos de forma y de contenido del lenguaje son las piezas fundamentales que cobran sentido en la recepción y expresión^(7)^, es decir, justifican que tal certeza de recepción o expresión tenga realmente elementos lingüísticos observables. Una calidad de evidencia baja (Cuadro 2.), en comparación a otros desenlaces, sugiere que la evaluación de estos aspectos no es confiable, sobre todo cuando se encuentra aislada, sin acompañarse de otros resultados como el lenguaje receptivo o expresivo. Se debe puntualizar que las herramientas de evaluación en los estudios seleccionados para esta área, adjuntos en el Anexo B, surgen de la apreciación tradicional - oral del lenguaje, por lo que resultan factibles de ser aplicadas cuando se obtienen datos producidos oralmente. Al considerar el estudio de adaptación de las herramientas para evaluación de lenguaje en niños signantes de Rodríguez-Ortiz et al.^(16)^, surge la necesidad de investigación y planteamiento de herramientas que evalúen elementos formales lingüísticos de la lengua de señas, por ejemplo, a partir de queremas^(17)^.

Vale la pena considerar los elementos de uso del lenguaje, pragmáticos^(7)^, en este apartado. A pesar de que únicamente son 2 los estudios que abordan este elemento (Cuadro 3.) es importante destacar que su evaluación, a pesar de incluirse en un nivel bajo de evidencia, toma en cuenta otros factores como el desarrollo cognitivo desde la Teoría de la Mente^(18,19)^, entendido más allá de las herramientas de evaluación de coeficiente intelectual. Estas últimas suelen valerse de ítems cuyo procesamiento implica, a priori, un manejo oral de lenguaje. Sin embargo, partir de otras consideraciones cognitivas, resultan valiosos en la construcción de nuevas formas de evaluación del desarrollo del lenguaje.

### La lengua oral frente a la lengua de señas

Las dos revisiones sistemáticas incluidas^(5,20)^, aunque se alejan de la dirección de sus conclusiones y priorizan dos intervenciones distintas, ponen en evidencia dos factores de vital importancia: el costo y recursos de los implantes y la duda en su efectividad a largo plazo, al no poder elevar el desarrollo del lenguaje del niño al aparente nivel estándar del niño oyente.

Los estudios que toman en cuenta la rehabilitación oral y el implante coclear, no adjuntan en sus resultados factores intervinientes sobre la posible presencia de gestos o incluso acercamientos a la lengua de señas. Esta situación seguramente se explica por los históricos prejuicios a la lengua de señas, donde se coloca a este idioma como un instrumento extra o incluso como un perjudicial estorbo de la habilidad oral^(20)^. La revisión sistemática de Hall^(20)^ es la única publicación en explicitar resultados sobre un desarrollo eficaz del lenguaje en general cuando hay implante sumado al uso lengua de señas (tal resultado lo obtiene del estudio del Davidson et al.^(21)^). Tal proeza se puede comparar con la breve anotación de la publicación de Meinzen-Derr et al.^(22)^ que mencionan que su estudio clínico aleatorizado, en población sordo oralizada, deben ser replicados, en un futuro, en población sordo signante.

### Otros aspectos a considerar

La mayoría de estudios concuerdan en que la edad de detección de la sordera, sumado a una intervención temprana, es capaz de responder mejor y oportunamente a las necesidades de los niños sordos; sin embargo, se distancian uno de otro en escoger el tipo de información, priorizando la rehabilitación oral, y dejando de lado a la lengua de señas.

En la gran mayoría de estudios se destaca un nivel de desarrollo del lenguaje, luego de la implantación coclear, cercano o similar al del niño oyente, más nunca igual. Se exceptúan aquellos que toman en cuenta la lengua de señas y los gestos, que ubican al niño sordo en mismo nivel que el oyente. Por lo tanto las posibilidades de la lengua de señas deben considerarse al momento de hablar de desarrollo y evaluación, incluso cuando es adquirida o aprendida por niños oyentes sin pérdida auditiva^(16)^. Tales consideraciones ponen en tela de juicio las revisiones y los estudios que no consideran en sus desenlaces los costos y recursos, además de los valores y preferencias de madres y padres con hijos sordos.

### Discusión de resultados:

La literatura publicada en relación a la eficacia de las intervenciones y la evaluación del lenguaje de niños sordos prelocutivos parece ser confiable si parten de la falta o reducción de la audición y la necesidad de habla oral^(23)^. Sin embargo, cuando el problema se aborda desde una óptica integral del lenguaje, las evidencias aún son escasas y no hay un consenso que permita determinar la mejor vía de evaluación por la cual optar. Esta cuestión seguramente se debe a un problema antecesor al de la evaluación: las opciones de intervención, o más bien, las decisiones de los profesionales médicos que, en su mayoría, abogan por un pronto ajuste del niño sordo a los estándares oyentes normativos. Desde ese punto de partida, la evaluación del lenguaje queda sesgada a los elementos observables que el niño sordo sea capaz de producir de forma oral, de los cuales se infiere la presencia de lenguaje. Sin embargo, estas aproximaciones dejan de lado la posibilidad de que el niño haya logrado algo de lenguaje con elementos que van más allá de la producción del sonido. Trayendo a colación ese espectacular cambio de paradigma de la interacción humana con las propuestas de Paul Watzlawick et al.^(24)^, se pone en contexto este “más allá de la producción de lenguaje” en el primer axioma de comunicación, “es imposible no comunicar”, que describe perfectamente el hecho de que, aunque un niño no produzca sonidos de habla, hay otras instancias de lenguaje que funcionan en su cabeza.

La detección e intervención temprana de la sordera es una necesidad urgente, y hay evidencia suficiente para colocarla como la mejor forma de prevención de problemas, no solo de lenguaje, en niños sordos. Sin embargo, considerando lo antes expuesto, es hora de empezar a provocar cambios de paradigmas extremos que permitan abordar al lenguaje de forma integral y en todas sus modalidades posibles. La presente revisión considera sumamente importante el valioso aporte que podría tener la participación de profesionales sordos, oralizados y signantes, tanto médicos como de otras áreas, en la producción de evidencia científica que tome en cuenta sus valores y preferencias. Sin duda, una aproximación tal, permitirá acortar las brechas tanto en las faltas de consenso mencionadas, como en las barreras de comunicación. Para ello, hace falta, no solo investigación, sino una mirada epistemológica de los tratamientos y resultados históricamente direccionados a la oralización.

Los dispositivos electrónicos para la sordera, implantes cocleares y audífonos, merecen una especial mención. La presente revisión, en ningún momento, tiene por objetivo desestimar, negar o prohibir su uso; al contrario, el interés se dirige a replantear la manera en que son ofrecidos y brindados a madres, padres y niños sordos. No se puede menospreciar el excelente crecimiento de la tecnología en los últimos años, y sería incompatible no sostener que seguramente los implantes mejorarán su alcance y sus beneficios. Sin embargo, frente a una acelerada evolución, más vale asegurarse de todos los factores intervinientes. Por un lado, es oportuno continuar construyendo evidencia sobre el impacto económico de los implantes cocleares para las familias^(5)^, por otro lado, empezar a describir, de forma integral una vez más, los efectos adversos a largo plazo, por ejemplo, el aparecimiento de hueso nuevo o la pérdida de los restos auditivos^(25)^.

Con el recorrido realizado en el momento, es pertinente mencionar la necesidad urgente de la transmisión del conocimiento a todos aquellos profesionales de la sordera que determinan el destino de los niños sordos. Tomar en cuenta los valores y preferencias de estos pacientes, no es sólo una cuestión médica, sino una problemática social y cultural que se enlaza directamente con la lucha que mantiene la Comunidad Sorda a nivel mundial. Por lo tanto, es necesario garantizar la capacitación completa y continua en temas de pérdida auditiva, sordera, comunidad sorda, cultura sorda, y lengua de señas, que aseguren ese abordaje integral que tanto se anhela. Con esos cambios, no solamente se espera un impacto en la intervención, sino en todo el aparato normativo, que es también responsable del destino del niño sordo.

Es importante mencionar que los estudios clínicos, podrían verse afectados por un sesgo de imprecisión en vista de que sus muestras son relativamente pequeñas, pero cuentan con buenos diseños y metodologías. Se debe considerar que la diversidad de la sordera es demasiado amplia, por lo que estos estudios se han esforzado por obtener muestras homogéneas. Aun así, se ha notado una falta de diferenciación entre las poblaciones que usan implante u audífono, o quienes tienes familia sorda u oyente, cuestiones que han disminuido la calidad de la evidencia relacionada al lenguaje expresivo. La presente revisión, debe reconocer que puede verse afectada por un sesgo de evidencia indirecta, en vista de que los estudios seleccionados toman en cuenta varios grados de pérdida auditiva, de leve a profunda, cuando se considera que el desarrollo de lenguaje será particular en cada uno. Además, las menciones al uso de implantes cocleares y audífonos deben considerarse como comentarios extras, en vista de que no formar parte de la pregunta de investigación. Sin embargo, se espera que sean invitaciones para futuras investigaciones y revisiones sistemáticas.

## CONCLUSIONES

Sobre la base de las calificaciones de la calidad del cuerpo de la evidencia de la evaluación del desarrollo en niños sordos prelocutivos, se considera que hay suficiente información confiable para realizarla durante los seis primeros años de vida. Sin embargo, la interpretación de sus resultados debe tomar en cuenta que los datos obtenidos sobre un desarrollo general del lenguaje deben sostenerse en mayor medida en evaluaciones de lenguaje receptivo sobre el expresivo, y que cuyas evidencias pueden enriquecerse al adjuntar evaluaciones de elementos lingüísticos formales tanto de la modalidad oral como gestual, y de los componentes pragmáticos de los procesos comunicacionales.

Se requiere más investigación sobre la evaluación de elementos formales de la modalidad gestual del lenguaje, las lenguas de señas, que permitan la construcción de herramientas (inventarios, escalas, protocolos de observación), capaces de contrastar los resultados del desarrollo de niños sordos según la intervención a la que hayan sido expuestos o la elección que hayan tomado.

## Anexo A. Estrategia y expresiones de búsqueda en principales fuentes de información

**Table:**

**BÚSQUEDA REALIZADA. Fecha de búsqueda: 08-02-2022**					
**N°**	**PLATAFORMA**	**EXPRESIÓN DE BÚSQUEDA**	**FILTROS**	**RESULTADOS**	**RESULTADOS CON CRITERIOS DE INCLUSIÓN Y EXCLUSIÓN**
1	PubMed	**(Child AND (Deafness OR Hearing Loss)) AND Language Development AND (Language Evaluation OR Evaluation OR Test)**	Niño: nacimiento-18 años	293	18
			Recién nacido: nacimiento-1 mes		
			Bebé: nacimiento-23 meses		
			Bebé: 1-23 meses		
			Niño Preescolar: 2-5 años		
			Últimos 5 años		
2	BVS-LILACS	**(Child AND (Deafness OR Hearing Loss)) AND Language Development AND (Language Evaluation OR Evaluation OR Test)**	Asunto principal: sordera, desarrollo del lenguaje	11	1
			Últimos 5 años		
3	BVS-IBECS	**(Child AND (Deafness OR Hearing Loss)) AND Language Development AND (Language Evaluation OR Evaluation OR Test)**	Asunto principal: sordera, desarrollo del lenguaje	3	0
			Últimos 5 años		
4	Clinical Trial	**(Child AND (Deafness OR Hearing Loss)) AND Language Development AND (Language Evaluation OR Evaluation OR Test)**	Age: 6 years	28	0
5	Trip Medical Data Base	**P(Child, Deafness) I (Language Development)**	Since 2017	11	2
6	Cochrane Library	**Deafness AND Child AND Language evaluation**	-	37	2
7	National Institute for Health and Care Excellence NICE	**Deafness**	-	20	1
**TOTAL**				**403**	**24**
**ELIMINACIÓN DE DUPLICADOS**					**21**
**SEGUNDA SELECCIÓN DE ARTÍCULOS PARA REVISIÓN (** **FIGURA 1** **.)**					**15**

## Anexo B. Estudios seleccionados para la revisión

**Table:**

N°	Autor	Título	Pacientes edad	Tipo de sordera e intervenciones	Evaluación de lenguaje relacionada	Resultados relacionados	D^1^
Revisiones Sistemáticas y Evaluaciones de Tecnología Sanitaria RS/ETS							
1	Ontario Health Quality^(5)^	Implantable Devices for Single-Sided Deafness and Conductive or Mixed Hearing Loss: A Health Technology Assessment	#NR^2^	Sordera unilateral, conductiva y mixta	Speech Intelligibility Rating (SIR)	Mejora en Desarrollo del lenguaje.	DL^3^
			Adultos	Implante coclear		Alto costo y recursos en el proceso posterior a la implantación coclear.	
			Niños				
2	Hall^(20)^	What You Don't Know Can Hurt You: The Risk of Language Deprivation by Impairing Sign Language Development in Deaf Children	#NR	Sordera en primeras etapas del desarrollo	Introducción de lengua de señas, lengua oral o ambas para un desarrollo del lenguaje de niños sordos.	Evidencia limitada sobre eficacia de lengua de señas como primera lengua.	O^4^
			Niños sordos en general			Intervención médica se dirige a niños sordos como “personas con problemas de audición”. Oralismo como forma de prevención de la exposición a la lengua de señas.	
						Retrasos de lenguaje afectan el desarrollo de estructuras cerebrales.	
						El implante coclear no garantiza el desarrollo del lenguaje a los dos años. Lo hace en niños que signan desde el nacimiento.	
						La privación del lenguaje parece ser la causa de los malos resultados de lenguaje de personas sordas.	
Ensayos Clínicos Aleatorizados ECA							
3	Meinzen-Derr et al.^(22)^	A Technology-Assisted Language Intervention for Children Who Are Deaf or Hard of Hearing: A Randomized Clinical Trial	Intervención: 21 niños sordos de 3 a 12 años.	Pérdida auditiva bilateral de leve a profunda	Longitud de frases al expresarse	Aumento en puntuaciones TALI vs TAU:	LR^5^
			Control: 20 niños sordos	Implante y audífonos	Sintaxis MLU	MLU (β = 0,91 frente a 0,15, *P* < 0,0001)	LE^6^
				Intervención:	Semántica NWD	NWD (β = 1,21 frente a 0,26, P = 0,005)	O
				TALI: niños con intervención de lenguaje asistida por tecnología	Discurso MTL	MTL (β = 11,04 frente a 2,65, *P* = 0,007)	
				Control:	Evaluación clínica de los fundamentos del lenguaje	Aumento en puntuación media	
				TAU: niño con tratamiento habitual	Puntuaciones lenguaje receptivo	Receptivo:	
					Puntuaciones lenguaje expresivo	TALI: 80,0 a 90,6 (p=0,008)	
						TAU: 82,1 a 83,6 (p=0,09)	
						Expresivo.	
						TALI: 77,6 a 86,1 (p=0,01)	
						TAU:77,5 a 79,9 (p= 0,21)	
				
4	Monshizadeh et al.^(26)^	The effectiveness of a specifically-designed language intervention protocol on the cochlear implanted 2. children's communication development	Intervención (I): 26 niños de 20 a 24 meses	Usuarios de implante Coclear IC,	Escalas de Bayley de Desarrollo de Infantes y niños pequeños- Tercera Edición.	Puntuación media (desviación estándar),	LR
			Control (C): 25 niños	Intervención: rehabilitación auditivo verbal e intervención basada en la cognición	Desarrollo General DG	DG:	LE
				Control: rehabilitación auditivo verbal.	Comunicación receptiva RE	I: 91 (10,73)	
					Comunicación expresiva EX	C:73,64(8,7)	
						Correlación de ambos grupos: t=6,33 df=49, (p=0,001)	
						RE: Correlación de ambos grupos (p=0,001)	
						EX: Correlación de ambos grupos (p=0,01)	
Estudios de Cohorte							
5	Yoshinaga-Itano et al.^(18)^	Early Intervention, Parent Talk, and Pragmatic Language in Children With Hearing Loss	124 niños de 4 a 7 años	Pérdida auditiva bilateral, leve a profunda	Lista de verificación de pragmática.	Puntuaciones medias (desviación estándar), valor P, e intervalo de confianza.	O
					Predicción de pragmática por edad cronológica EC	EC 0,15 (0,01) p<0,01 (7,73 - 8,90)	
					Predicción de pragmática por el coeficiente intelectual CI	CI 0,08 (0,01) p<0,01 (0,05 - 0,11)	
					Predicción de pragmática por la educación de las madres EM	EM 0,23 (0,10) p<0,17 (0,04 - 0,42)	
					Predicción de pragmática por el número de palabras de los padres por minuto PP	PP 0,04 (0,01) p<0,003 (0,02 - 0,07)	
					Predicción de pragmática por el grado de pérdida GP	GP -1,08 (0,50) p<0,34 (-2,07 - 0,08)	
					Predicción de pragmática por cumplir EHDI	EHDI 1,00 (0,49) p<0,043 (0,03 - 1,97)	
6	Li et al.^(27)^	Developmental performance among pediatric candidates for cochlear implantation	500 niños de 6 a 72 meses	Hipoacusia severa profunda	Habilidades de desarrollo de Gesell	Retraso en desarrollo general (p< 0,001)	DL
				Candidatos a implante Coclear IC	Desarrollo general	Retraso en habilidades verbales y no verbales (p<0,05)	
					Desarrollo del lenguaje	El desarrollo del lenguaje fue el peor (tasa normal 4,2%)	
Estudios de Casos y Controles							
7	Guo and Spencer^(28)^	Development of Grammatical Accuracy in English-Speaking Children With Cochlear Implants: A Longitudinal Study	10 niños sordos de 3 a 5 años	Sordera prelocutiva	Unidades de comunicación gramatical (PGCU o Unidades C), en contar historias.	Duración de experiencia auditiva	O
			Implante Coclear (IC)	Implante Coclear IC	Efecto significativo de la duración de experiencia auditiva en PGCU	F(2, 18) = 3.99, p = .037, η _p_^2^ = .31	
			Grupo control: 10 niños con audición normal de 3 a 5 años		Producción de PGCU de niños con IC más baja que niños con audición normal a los 3 años de implantación	Producción de PGCU:	
					Producción de PGCU de niños con IC más baja que niños con audición normal a los 4 años de implantación	A 3 años:	
					Producción de PGCU de niños con IC más baja que niños con audición normal a los 5 años de implantación	F(1, 18) = 1,75, **p = 0,20**, η _p_^2^ = 0,09	
						A 4 años:	
						F(1, 18) = 4.78, p = .04, η _p_^2^ = .21	
						A 5 años:	
						F(1, 18) = 5.74, p = .03, η_p_^2^ = 0,24	
8	Wie et al.^(29)^	Long-Term Language Development in Children With Early Simultaneous Bilateral Cochlear Implants	19 niños de 5 a 18 meses	Sordera profunda congénita	Habilidades lingüísticas en 10 momentos	Niños con IC en comparación a audición normal. Diferencias significativas según los meses después de la implantación.	DL
			Grupo control: 19 niños con audición normal	Implantes Cocleares bilaterales IC	Subescala de Comprensión-Conceptual del cuestionario para padres del Inventario de Desarrollo Infantil de Minnesota (MCDI)	DGL:	LR
					Desarrollo general del lenguaje DGL	Antes de 12: sin diferencias significativas.	LE
					Escala Mullen de Aprendizaje Temprano (MSEL)	12 meses: *p* = 0,004; *d* = 1,21	
					Lenguaje general receptivo RE	A partir de 36 meses: sin diferencias significativas	
					Lenguaje general expresivo EX	RE:	
					Escala británica de vocabulario pictórico, segunda edición (BPVS II)	3 meses: *p* < 0,001; *d* = 2,56	
					Vocabulario receptivo VRE	36 meses: sin diferencias significativas	
					Subprueba de denominación de imágenes de la Wechsler Preschool and the Primary Scale of Intelligence, tercera edición (WPPSI-III)	EX:	
					Vocabulario expresivo VEX	3 meses: *p* < 0,001; *d* = 2,51	
						48 meses: sin diferencias significativas.	
						VRE:	
						36 meses: sin diferencias significativas	
						48 meses: *p* = 0,02; *d* = 0,88	
						60 meses: *p* < 0,001; *d* = 1,53	
						72 meses: *p* < 0,001; *d* = 1,36	
						VEX:	
						Sin evidencias significativas	
9	Werfel^(30)^	Morphosyntax Production of Preschool Children With Hearing Loss: An Evaluation of the Extended Optional Infinitive and Surface Accounts	18 pre-escolares de 45 a 6 meses	Sordera moderada a profunda	Protocolo de Hadley.	Prueba de X2: el lenguaje espontáneo no difiere del tipo de amplificación (p>0,05)	O
			Grupos control, audición normal	Implantes cocleares y audífonos	Morfemas de Brown.	Puntuaciones medias en tres grupos: sordos - control 1 - control 2:	
			1. Misma edad(18)		Subescala de lenguaje expresivo - Test of Early Language Development-Third Edition (TELD-3)	MLU: 4,37 - 5,82 - 5,22 (p=0,25)	
			2. Mismo idioma(18)		Morfemas, longitud de la frase MLU	NDW: 146,44 - 183,50 - 173,33 (p=0.065)	
					Semántica, número de palabras diferentes NDW	TNW: 413,22 - 525,89 - 497,72 (p=0,174)	
					Número total de palabras TNW		
10	Netten et al.^(19)^	Can You Hear What I Think? Theory of Mind in Young Children With Moderate Hearing Loss	44 niños sordos de 3 a 5 años	Pérdida auditiva, preferían usar el lenguaje hablado	Reynell Developmental	Correlaciones de niños sordos (Partial r)	LR
			Grupo control: 101 niños oyentes		Language Scale, Schlichting	De niños, ToM y RE:	LE
					Expressive Language test y Escalas para padres	Similar desire: 0,36 (p<0,05)	
					Obtenido el niño N:	Dissimilar desire: 0,24 (p>0.05)	
					Correlación de Lenguaje receptivo RE y expresivo EX con aspectos de la teoría de la mente ToM, en comparación con el grupo control.	False belief: 0.56 (p<0,001)	
					Obtenido de los padres P:	De niños, ToM y EX:	
					Correlación de comprensión del lenguaje CL y expresión del lenguaje EL con aspectos de la teoría de la mente, en comparación con el grupo control.	Similar desire: 0.32 (p<0.05)	
						Dissimilar desire: -0.01	
						False belief: 0.35 (p<0,05)	
						De padres ToM y CL:	
						Similar desire: 0,09 (diferencia significativa)	
						Dissimilar desire: 0.26 (p<0,01)	
						False belief: 0.24 (p<0,01)	
						De padres ToM y EL:	
						Similar desire: 0,22 (p>0.05)	
						Dissimilar desire: 0,13 (p>0.05)	
						False belief: 0,29 (p<0,001)	
11	Aslıer et al.^(31)^	The influence of age and language on developmental trajectory of theory of mind in children with cochlear implants	111 niños de 36 a 132 meses	Sordera profunda bilateral congénita	Sally-Ann (prueba de Teoría de la Mente ToM)	Las puntuaciones altas en lenguaje se relacionan con un mejor rendimiento en la prueba de Sally-Ann (p<0,05)	O
			Grupo control: más de 99 niños	Implante Coclear IC	Peabody picture vocabulary		LR
					Lenguaje receptivo		
Estudios Observacionales							
12	Scarabello et al.^(32)^	Language evaluation in children with pre-lingual hearing loss and cochlear implant.	30 niños de 36 a 72 meses	Hipoacusia neurosensorial bilateral severa y profunda.	ABFW Child Language Test-Part B - Vocabulary. Lenguaje oral expresivo.	Correlación de factores con puntuaciones de ABFW: diferencias significativas y valores p por cada elemento de la prueba. Remitirse a artículo.	O
				Implante Coclear IC	Test de Peabody Picture. Lenguaje oral receptivo	Correlación de factores con puntuaciones de test Peabody:	
						Edad de cirugía: −0.297 (p=0.168)	
						Edad de evaluación: −0.645 (p= 0.001)	
						Tiempo de uso de implante: −0.332 (p= 0.122)	
						Fonemas: 0.020 (p= 0.929)	
						Palabras: 0.012 (p= 0.957)	
13	Kutlu et al.^(33)^	A study on the association of functional hearing behaviors with semantics, morphology and syntax in cochlear-implanted preschool children	48 niños de 3 a 5 años con 11 meses.	Pérdida auditiva bilateral severa y profunda	Test de Desarrollo Temprano del Lenguaje - Tercera Edición (TELD-3)	Relación entre semántica, morfología, sintaxis, y la habilidad auditiva funcional (p<0,05)	DL
				Implante Coclear IC	Lenguaje receptivo	Correlación entre lenguaje receptivo, lenguaje receptivo semántico y lenguaje receptivo gramático con comunicación verbal: (r = 0.781; r = 0.729; r = 0.787) (0.627; p < 0.01)	LR
					Lenguaje expresivo	Correlación entre lenguaje expresivo, lenguaje expresivo semántico y lenguaje expresivo gramático con comunicación verbal	LE
					Formulario de Funcionamiento después de la implantación Coclear Pediátrica (FAPCI)	(r =0.797; r = 0.749; 0.782) (0.757; p < 0.01)	
Otros estudios y publicaciones							
14	Rodríguez-Ortiz et al.^(16)^	A Spanish Sign Language (LSE) Adaptation of the Communicative Development Inventories	55 niños de 8 a 36 meses	Sordos signantes	Adaptación del Inventario de Desarrollo Comunicativo de MacArthur (CDI) Fenson et al., 1993.	Comprenden más señas de las que producen. Edad más baja y más alta:	LR
					Comprensión de señas en comparación de las producidas	A los 8-11 meses, *z*(16) = 3,52, *p <*, 001, η *^2 =^* 12,686;	LE
					Lenguaje receptivo y expresivo	A los 32-36 meses, *z*(31) = 4,83, *p <* 0,001, η *^2 =^* 12,309.	
						El desarrollo de lenguaje receptivo y expresivo es similar	
						A los 8-11 meses, *r*(16) = 0,64, *p =* 0,008.	
						A los 32-36 meses, *r*(31) = 0,81, *p <* 0,001.	
15	Goldin-Meadow^(34)^	Using Gesture To Identify and Address Early Concerns About Language and Pragmatics	Niños sordos en general	Niños que usan gestos antes de palabras o señas	Observación de gestos (no estandarizados - señas) para detectar problemas del desarrollo y pragmática.	NR	O
						Sugiere posible efectividad	

1 D: desenlaces incluidos

2 NR: no reporta datos

3 DL: Publicación incluida en el desenlace Desarrollo general del lenguaje

4 O: Publicación incluida en el desenlace Otros elementos del lenguaje

5 LR: Publicación incluida en el desenlace Lenguaje Receptivo

6 LE: Publicación incluida en el desenlace Lenguaje Expresivo
